# Pathotyping Citrus Ornamental Relatives with *Xanthomonas citri* pv. *citri* and *X. citri* pv. *aurantifolii* Refines Our Understanding of Their Susceptibility to These Pathogens

**DOI:** 10.3390/microorganisms10050986

**Published:** 2022-05-08

**Authors:** Grazia Licciardello, Paola Caruso, Patrizia Bella, Claudine Boyer, Malcolm W. Smith, Olivier Pruvost, Isabelle Robene, Jaime Cubero, Vittoria Catara

**Affiliations:** 1 Dipartimento di Agricoltura Alimentazione e Ambiente, Università degli Studi di Catania, 95130 Catania, Italy; grazia.licciardello@crea.gov.it; 2 Centro di Ricerca Olivicoltura, Frutticoltura e Agrumicoltura-Consiglio per la Ricerca in Agricoltura e L’analisi Dell’Economia Agraria (CREA), 95024 Acireale, Italy; paola.caruso@crea.gov.it; 3 Dipartimento di Scienze Agrarie, Alimentari e Forestali, Università degli Studi di Palermo, 90128 Palermo, Italy; patrizia.bella@unipa.it; 4 CIRAD, UMR Peuplements Végétaux et Bioagresseurs en Milieu Tropical (PVBMT), 97410 Saint Pierre, La Réunion, France; claudine.boyer@cirad.fr (C.B.); olivier.pruvost@cirad.fr (O.P.); isabelle.robene@cirad.fr (I.R.); 5 Department of Agriculture & Fisheries, Bundaberg Research Station, Bundaberg, QLD 4670, Australia; malcolm.smith@daf.qld.gov.au; 6 Departamento de Protección Vegetal, Instituto Nacional de Investigación y Tecnología Agraria y Alimentaria/Consejo Superior de Investigaciones Científicas, 28040 Madrid, Spain; cubero@inia.csic.es

**Keywords:** citrus bacterial canker, Rutaceae, ornamental plants, hyperplastic tissue, pathotype, host–plant interaction

## Abstract

*Xanthomonas citri* pv. *citri* (*Xcc*) and *X. citri* pv. *aurantifolii* (*Xca*) are causal agents of Citrus Bacterial Canker (CBC), a devastating disease that severely affects citrus plants. They are harmful organisms not reported in Europe or the Mediterranean Basin. Host plants are in the Rutaceae family, including the genera *Citrus, Poncirus*, and *Fortunella*, and their hybrids. In addition, other genera of ornamental interest are reported as susceptible, but results are not uniform and sometimes incongruent. We evaluated the susceptibility of 32 ornamental accessions of the Rutaceae family belonging to the genera *Citrus, Fortunella, Atalantia, Clausena, Eremocitrus, Glycosmis, Microcitrus, Murraya, Casimiroa, Calodendrum*, and *Aegle*, and three hybrids to seven strains of *Xcc* and *Xca*. Pathotyping evaluation was assessed by scoring the symptomatic reactions on detached leaves. High variability in symptoms and bacterial population was shown among the different strains in the different hosts, indicative of complex host–pathogen interactions. The results are mostly consistent with past findings, with the few discrepancies probably due to our more complete experimental approach using multiple strains of the pathogen and multiple hosts. Our work supports the need to regulate non-citrus Rutaceae plant introductions into areas, like the EU and Mediterranean, that are currently free of this economically important pathogen.

## 1. Introduction

Since the early days of plant pathology, research studies have predominantly focused on economically important crops, and plant pathogenic bacteria are no exception. Even on extensively studied pathosystems, host plants with little or no economic significance have received much less attention. A refined understanding of plant disease emergences requires more research to be conducted on them, as they can constitute (i) epidemiologically significant reservoirs of generalist pathogens (e.g., *Ralstonia pseudosolanacearum*, *R. solanacearum* or *Xylella fastidiosa*), (ii) biological models of choice for a refined understanding of species jump, and (iii) uncontrolled pathways for long distance movement of regulated pathogens [1,2,3,4].

*Xanthomonas citri* pv. *citri* (*Xcc*), and *Xanthomonas citri* pv. *aurantifolii* (*Xca*) are causal agents of Citrus Bacterial Canker (CBC), a devastating disease that severely affects citrus plants [5]. *Citrus*, *Poncirus*, *Fortunella*, and their hybrids are the most common natural host genera [6]. In addition, natural infections have been described in *Atalantia buxifolia, Casimiroa edulis, Citropsis daweana, Clausena harmandiana, Eremocitrus glauca, Microcitrus* spp., *Naringi crenulata, Swinglea glutinosa*, and *Zanthoxylum ailanthoides,* but incongruent data were sometimes reported [7,8,9,10,11].

EFSA [5] also stated that on the basis of the development of lesions after artificial inoculation, other plants such as *Acronichia* (*A. acidula*), *Aegle* (*A. marmelos*), *Aegelopsis* (*A. chevalieri*), *Atalantia* (*A. ceylonica*, *A. citrioides* and *A. guillauminii*), *Casimiroa* (*C. edulis*), *Clausena* (*C. lansium*), *Citropis* (*C. articulata*), *Eremocitrus* (*E. glauca*), *Feroniella* (*F. lucida*), *Limonia* (*L. acidissima*), *Lunasia* (*L. amara*), *Melicope* (*M.* denhamii and *M. triphylla*), *Microcitrus* (*M. australasica* and *M. garrowayae*), *Micromelum* (*M. minutum*), *Murraya* (*M. exotica*, and *M. ovatifoliata*), *Paramignya* (*P. longipedunculata* and *P monophylla*), *Tetrasium sp.*, *Toddalia* (*T. asiatica*), and *Zanthoxylum* (*Z. clava-herculis* and *Z. fagara*) may be considered susceptible to citrus canker bacteria [5]. However, most of the results on these species of ornamental interest were from studies performed a century ago when the existence of variants of the pathogen had not yet been reported. In addition, the most recent studies have been carried out conducting artificial inoculation using just one single strain or in natural conditions where it was assumed that pathotype A was the causal strain.

*Xcc* and *Xca* are classified as harmful organisms not reported in any part of Europe or the Mediterranean Basin, and whose introduction and spread within the EU shall be banned. These two pathovars cause the so-called Asiatic and South American citrus bacterial canker, respectively [5]. Within each pathovar, different pathogenic variants called pathotypes have been described—pathotype A includes A, A*, and A^W^ variants within the pv. *citri,* and pathotypes B and C in the *aurantifolii* pathovar. Symptomatology among different pathotypes is similar, although they differ in host range [12]. *Xcc* pathotype A is the most widespread CBC causal agent and has the largest host range [13]. Conversely, strains A* and A^w^, coming from Southwest Asia and Florida, respectively, cause the disease under natural conditions primarily on Mexican lime (*Citrus aurantiifolia*), and to a lesser extent sweet lime (*C. limettioides*), Tahiti or Persian lime (*C. latifolia*), and alemow (*C. macrophylla*) [12,14]. Comparative genomics of 42 strains of the three pathotypes of *Xcc* (A, A*, A^W^) concluded that they were monophyletic but showed multiple differences in gene content, putative pseudogenization, and mutations in genes involved in pathogenicity [15]. At an infra-pathotype level, extensive variation in gene-content has been detected, with plasmids constituting an important vehicle for horizontal gene transfer and acquisition of adaptive traits [16,17,18].

*Xca* was geographically restricted to Argentina, Paraguay, Uruguay, and Brazil, where it was described to cause B and C canker on lemon (*C. limon*) and Mexican or Key lime, respectively, although nowadays these bacteria are not associated with outbreaks in any area [13,19,20].

The precise evolutionary relationship among the three pathotypes A, A^w^, and A* is still questioned. The presence of an [A + A^w^] clade, observed by Gordon et al. [15], was confirmed by the analysis of a dataset of 95 *Xcc* genomes [21]. Moreover, from the above works it may be concluded that recombination in different genomic regions, especially in genes involved in virulence, could have had a significant role in the evolution of *Xcc* pathovars [15,21]. Comparative genomics also suggested a massive geographical expansion supported by worldwide import/export activities in the citrus industry [21].

Despite recent advances in pathogen taxonomy and evolution, available pathogenicity data on species of ornamental interest are mostly from studies performed a century ago when the existence of variants of the pathogen had not yet been reported. In addition, more recent studies have been carried out by conducting artificial inoculations using just one single strain or under natural conditions where it was assumed that only the pathotype A was present.

To address this topic, we have studied the response of 32 accessions from the Rutaceae family and three hybrids following inoculation with seven strains of *Xanthomonas citri*, including two pathovars, pv. *citri* and pv. *aurantifolii*, covering all the described pathotypes.

## 2. Materials and Methods

### 2.1. Bacterial Strains

*Xcc* and *Xca* strains used in this study are listed in Table 1 and were selected in order to have at least one strain per pathotype, taking into account the lineages described for the pathotypes A, A*, and A^W^, according to Pruvost et al. [22] and Gordon et al. [15]. Bacterial strains of the pathotypes A, A*, and A^W^ were preferentially cultivated on WB medium (Wilbrink medium, 5 g L^−1^ proteose peptone, 0.5 g L^−1^ K_2_HPO_4_, 0.3 g L^−1^ MgSO_4_, 10 g L^−1^ sucrose, 15 g L^−1^ purified agar), whereas strains of the B and C pathotypes were grown on SPA (Sucrose peptone agar 10 g L^−1^ sucrose, 5 g L^−1^ peptone, 0.6 g L ^−1^ K_2_SO_4_, 0.3 g L^−1^ MgSO_4_, 15 g L^−1^ purified agar) by incubation at 28 °C for 48 h. Bacterial strains were long-term stored in Nutrient Broth plus 20% glycerol at −80 °C.

### 2.2. Plant Material

Plant material used to assess host–pathogen interaction with *Xcc* and *Xca* was obtained from accessions from the germplasm collection at CREA-Research center for Olive, Fruit, and Citrus Crops (Acireale, Catania, Italy) and at CIRAD (La Reunion, France) (Table 2). Species in the genera *Aegle*, *Atalantia*, *Calodendrum*, *Casimiroa*, *Citrus*, *Clausena*, *Eremocitrus*, *Fortunella*, *Glycosmis*, *Microcitrus*, *Murraya*, and hybrids which are used as ornamental plants were included (Table 2). Three species, *Citrus aurantiifolia*, *C. paradisi*, and *Fortunella margarita*, were included as host–response reference plants because they had been reported as susceptible to all pathotypes, susceptible only to pathotype A and A*, or resistant, respectively [12,14].

### 2.3. Pathogenicity Test

**Figure 1:**
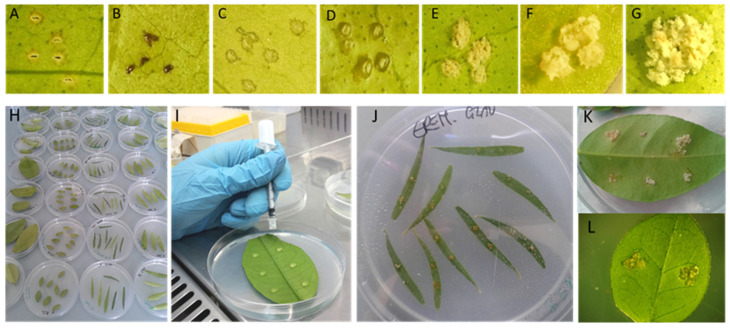
Host–bacterial strain interaction assessed by an in vitro test on detached leaves of Rutaceae inoculated with bacterial strains of *Xanthomonas citri* pv. *citri* and pv. *aurantifolii*. (**A**–**G**): exemplificative scale of symptomatic reactions measured as: “0”, wound repair (**A**) or necrosis (**B**); “ws”, water-soaked margin surrounding the wound sites (**C**); “+”, pustule or blister-like lesions (**D**); “++”, beginning of callus formation or crystalline callus at inoculation site (**E**); “+++”, abundant crystalline callus (5 sites) (**F**); “++++”, confluent crystalline callus in a single hyperplasia (**G**). (**H**) leaves of different Rutaceae placed on the surface of Petri dishes containing soft water agar (1%) with the abaxial side up; (**I**), wound inoculation with a sterile syringe needle (five wounds per site); (**J**–**L**), crystalline callus visible at 20 days post-inoculation on leaves of *Eremocitrus glauca* (**J**), *Citrus aurantiifolia* (**K**), and *Murraya ovatifoliolata* (**L**).

Young shoots 20–25 cm long were collected from plants maintained in greenhouses or in the open field, placed inside sealable plastic bags, moved to the laboratory, stored at 4°C, and used within 24–36 h. To evaluate the susceptibility of the different plant species, a detached leaf assay previously described was used [23]. Leaves were rinsed with tap water to remove impurities and moved to a Biohazard hood where they were surface sterilized, first by immersion in 70% ethanol for 30 s, then in a solution of 0.5% sodium hypochlorite for 30 s, and lastly rinsed three times with sterile distilled water [24]. For accessions with large leaves, at least three leaves were tested, whereas for small leaves accessions, 6–10 leaves were tested per treatment. Disinfected leaves were dried on sterile paper towels and placed on the surface of Petri dishes containing soft water agar (1%) with the abaxial side up. *Xcc* and *Xca* inoculums were prepared from culture plates in sterile distilled water adjusting the concentration to 0.1 OD at 600 nm (approximately 1 × 10^8^ cfu mL^−1^ as confirmed by dilution plating). Ten-µL drops of the bacterial suspensions were deposited on the leaf in 2–6 sites according to the leaf size and 5 wounds per drop were created with a sterile 25-gauge syringe needle (Figure 1). Negative controls were inoculated with sterile distilled water. Petri dishes were immediately sealed with Parafilm **^®^** M and incubated in a growth chamber at 28 °C (fluorescent light at 60 μmol m^−2^ s^−1^ with a 12-h photoperiods). Symptoms on the inoculated detached leaves were assessed 5, 10, 15, and 20 days post-inoculation (dpi). The experiment was repeated at least twice, which made a total of at least 24 replicates on six different leaves for each strain–host combination. Symptoms per inoculation site were counted under a stereo microscope at 15–40× magnification.

Host–bacterial strain interaction was assessed visually, and scored using a six-point scale as follows: “0”, no symptoms as per water (negative control) inoculations, usually wound repair, and/or necrosis; “ws”, water-soaked margin surrounding the wound sites; “+”, swelling of cell evident at site of inoculation (pustule or blister-like lesions); “++”, beginning of callus formation or crystalline callus at inoculation site; “+++”, abundant crystalline callus (at the 5 sites); “++++”. Confluent crystalline callus in a single hyperplasia (Figure 1).

### 2.4. Estimation of Bacterial Population Sizes

The bacterial population size was assessed in 13 species at the inoculation site, 7 dpi. Two leaf disks (6 mm diameter), circumscribing the inoculated sites, were excised using a cork borer and surface sterilized in 0.5% sodium hypochlorite for 30 s, washed twice in sterile distilled water, and ground in 1.0 mL of sterile distilled water in 1.5 mL Eppendorf tubes using a sterile plastic pestle. Three replicates were used for each plant–bacterium combination. Ten-µL of serial dilutions of bacterial macerate in sterile distilled water were plated on kasugamycin-cephalexin-chlorothalonil (KCC; nutrient agar plus kasugamycin 16.0 mg L^−1^, cephalexin 16.0 mg L^−1^, and cycloheximide 100 mg L^−1^) agar medium [25]. Bacterial colonies were counted after 48 h and population size expressed as Log cfu per inoculation site. Data were analyzed by ANOVA using STATGRAPHICS Plus 5. Mean values were compared using the Student–Newman–Keuls test. All plastic and specimens used in the experiments were discarded as biohazard waste.

## 3. Results

### 3.1. Assessment of the Detached Leaf Assay

The detached leaf assay was first set up with the reference plant species *C. aurantiifolia*, *C. paradisi* and *F. margarita*, already investigated for their response to the different pathotypes of *Xcc* and *Xca* [12,14]. Detached leaves were inoculated with the seven strains (three pathotype A strains and one strain each for the pathotype A*, A^w^, B, and C) (Table 1). *C. aurantiifolia* developed typical CBC symptoms with all strains, *C. paradisi* with only A and A* strains, and *F. margarita* developed no symptoms (Table 3). Symptoms appeared earliest on *C. aurantiifolia* inoculated with *Xcc* LG97 (A) and *Xca* JV596 (C) as white crystalline callus developing from each pin wound (5 dpi), which, after 10 dpi, merged in a unique hyperplasia (data not shown). By 10 dpi, symptoms evolved drastically with the presence of white crystalline callus and cell hyperplasia with all *Xcc* and *Xca* strains, except for *Xca* JJ159 (B) in which the white callus appeared 15 dpi (Table 3).

At 5 dpi, inoculation of *C. paradisi* had resulted in callus formation with *Xcc* C40 strain (A) and swelling and water soaking with remaining pathotype A strains: *Xcc* LG97, and *Xcc* LE116 (A), and *Xcc* LG115 (A*) (data not shown). By 10 dpi, leaves inoculated with A and A* strains induced the formation of erumpent white callus, which evolved in advanced lesions tending to brown callus only with *Xcc* C40 and *Xcc* LG97. In contrast, Xcc LG115 (A^W^) induced hypersensitive response, whereas only wound repair was induced by *Xca* JJ159 (B) and *Xca* JV596 (C). No symptoms were observed on *F. margarita* leaves inoculated with any of the 7 bacterial strains.

**Table 2:** Ornamental rutaceous plant accessions used for characterization of the host range and their reaction to the *Xanthomonas citri* pv. *citri* (pathotype A, A*, and A^W^) and *Xanthomonas citri* pv*. aurantifolii* (pathotypes B and C).

Accession Number *^1^*	Botanical Name	Common Name	Synonyms
CREASSGCF1P5	*Aegle marmelos* (L.) Correa	Indian bael fruit	*Crataeva marmelos* (L.) sp. Pl.
CREASSVC	*^2^ Atalantia buxifolia* (Poir.) Oliv.	Chinese box orange	*Severinia buxifolia* (Poir.) Oliv.
SRA746	*^2^ Atalantia buxifolia* (Poir.) Oliv.	Chinese box orange	*Severinia buxifolia* (Poir.) Oliv.
SRA745	*Atalantia ceylanica* (Arn.) Oliv.	Ceylon atalantia	*Rissoa ceylanica* (Arn.).
CREASSGCF5P35	*^2^ Atalantia disticha* (Blanco) Merr.	Philippine box orange	*Severinia disticha* (Blanco)
SRA1088	*Balsamocitrus dawei* (Stapf.)	Uganda powder flask	--
CREASSGCF4P4	*Calodendrum capense* (Thunb.)	Cape chestnut	*Tarenna papyracea* Burtt Davy
CREASSGCF6P13	*Casimiroa edulis* (La Llave and Lex.)	White sapote;	--
CREASSGCF5P9	*Clausena excavata* (Burm. f.)	Pink wampee	*Murraya burmanni* (Spreng.)
CREASSGCF6P2	*Clausena lansium* (Lour.) Skeels	Wampee	*Cookia punctata* (Sonner)
SRA1080	*Clausena lansium* (Lour.) Skeels	Wampee	*Cookia punctata* (Sonner)
CREASSGCF46P8	*Citrus aurantiifolia* (Christm.) Swingle	Mexican lime	*Limonia aurantiifolia* (Christm.)
CREASSGCF37P4	*^3^ Citrus limonia* Osbeck var. *limonia*	Borneo red Rangpur lime;	--
CREASSGCF37P8	*^4^ Citrus microcarpa (Bunge)*	Calamondin;	*Citrus madurensis* (Lour.), *Citrus mitis* (Blanco)
CREASSGCF31P8	*Citrus myrtifolia* (Raf.)	Myrtle-leaf orange; Chinotto	*Citrus aurantium var. myrtifolia*
CREASSGCF23P12	*Citrus paradisi* (Macfad.).	Grapefruit	*Citrus decumana* var*. racemosa* (Roem.)
CREASSGCF35P1	*Citrus wintersii* (Mabb.)	Brown River finger lime	*Microcitrus papuana (Winters).*
CREASSGCF8P1	*^5^ Eremocitrus glauca* (Lindl.) Swingle	Australian desert lime	*Triphasia glauca* (Lindl.)
SRA871	*^5^ Eremocitrus glauca* (Lindl.) Swingle	Australian desert lime	*Triphasia glauca* (Lindl.)
SRA1001	*^5^ Eremocitrus glauca* (Lindl.) Swingle	Australian desert lime	*Triphasia glauca* (Lindl.)
CREASSGCF38P3	*^5^ Fortunella hindsii* (Champ. ex Benth.) Swingle	Hong Kong wild kumquat	*Sclerorostylis hindsii* (Champ. ex Benth. Hook.)
CREASSGCF8P10	*^5^ Fortunella japonica* (Thunb.) Swingle	Round kumquat	*Citrus japonica* (Thunb.)
CREASSAP	*^5^ Fortunella margarita* (Lour.) Swingle	Oval kumquat	*Citrus margarita* (Lour.)
SRA490	*^5^ Fortunella margarita* (Lour.) Swingle	Nagami kumquat	*Citrus margarita* (Lour.)
CREASSGCF20P4	*^5^Fortunella obovata* (Hort. ex Tanaka)	Fukushu kumquat	--
CREASSGCF10P2	*Glycosmis pentaphylla* (Retz.) DC.	Orangeberry	*Glycosmis cochinchinensis*
SRA1002	*^5^ Microcitrus australasica*	Australian finger lime	*Citrus australasica* (F. Muell.)
CREASSGCF38P6	*^5^ Microcitrus australis* (Planch.) Swingle	Australian round lime	*Limonia australis* (A. Cunn.)
CREASSGCF5P12	*Murraya koenigii* (L.) Spreng.	Curry leaf	*Bergera koenigii* (L. Mant. Pl.)
CREASSGCF22P2	*^6^ Murraya ovatifoliolata* (Engl.) Domin	--	*Murraya paniculata* var. *ovatifoliolata* Engl.
CREASSGCF36P8	*Murraya paniculata* (L.) Jack, Malay	Orange jasmine	*Chalcas paniculata* (L.) Mant. Pl.
SRA906	*Swinglea glutinosa* (Blanco) Merr.	Tabog	*Chaetospermum glutinosum* (Blanco) Swingle
	Hybrids	Parentage/origins	Synonyms
CREASSGCF2P3	Citrangequat v. Thomasville	Trigeneric hybrid [*Fortunella* sp. × (*Citrus sinensis* × *Poncirus trifoliata*)]	*Citrus* × *insitorum* Mabb × *Fortunella margarita*
CREASSGCF5P4	Faustrimedin	Trigeneric hybrid of three genera: *Citrus*, *Microcitrus* and *Fortunella*.	
CREASSGCF12P3	Limequat’ Lakeland’	Intergeneric hybrid between *Citrus aurantiifolia* × *Fortunella japonica.*	

***^1^*** CREA: CREA—Research center for Olive, Fruit, and Citrus Crops, Acireale, Catania, Italy; SRA: CIRAD INRAE CRB Citrus, San Giuliano, Corsica, France. ***^2^*** The genus *Atalantia* has a puzzling position in the phylogeny of the Citreae. It was grouped with *Severinia* [26]. In a study performed by Centre for Australian National Biodiversity Research, all phylogenetic trees obtained, apart from that of the trnL intron and spacer region, showed that *Severinia buxifolia* and *Atalantia ceylanica* appeared as a monophyletic clade. Originally part of the *Atalantia* genus, five of the six *Severinia* species were only segregated from *Atalantia* in 1938 by Swingle but are morphologically very similar to *Atalantia*. With sequences of only four of the eleven *Atalantia* species and one *Severenia*, our results merely highlight the need for further work on these genera (https://www.anbg.gov.au/cpbr/summer-scholarship/2004-projects/rich-citrus-2004/index.html, accessed on 3 March 2021). *^**3**^ C*. × *limonia* includes all *C. reticulata*/*C. medica* admixture types and, particularly according to Curk et al. [27] and Wu et al. [28], the direct hybrids between these two species: *C*. × *limonia* var. *limonia* (“Rangpur,” “Karna,” “Khatta,” “Khatta Karna” limes) [6]. ***^4^*** The exact hybrid nature of the calamondin remains to be established. It is commonly accepted to be a hybrid of a sour mandarin type and a kumquat. The most frequently mentioned candidates are the sour mandarin *Citrus sunki* (Tanaka) of 1927 (which is the later *Citrus reticulata* var. *austera* of Swingle of 1942) and the Oval or ‘Nagami’ kumquat *Citrus japonica* (Thunb.). ***^5^*** Based on the recent phylogenomic data and biological characteristics (particularly sexual compatibility), Ollitrault et al. [6] proposed the inclusion of the genera *Microcitrus*, *Eremocitrus*, *Clymenia*, *Poncirus*, and *Fortunella* in the *Citrus* genus as described by Mabberley [29,30]. However, to avoid confusion, and also because some other aspects regarding the specific subdivisions delimitations within the *Citrus* genus and the origin of admixture types proposed by Mabberley are not in agreement with recent molecular studies and its classification system is still incomplete, we refer to these species with Swingle and Reece classification [31]. ***^6^*** Reduction of *Murraya ovatifoliolata* and *M. paniculata* cv ‘Exotica’ to synonymy of *M. paniculata* is not accepted by Queensland botanists [32].

### 3.2. Plant Host–Bacterial Strain Interaction Phenotype

Variable results were obtained following inoculation of the seven strains in the detached leaves of the thirty-two ornamental rutaceous plants. The formation of hyperplastic tissue at the inoculation point, either blister like (pustules) or crystalline callus, although of different entities, was recorded as positive inoculation (the interaction phenotype scale is described in material and methods and in Table 3; Figure 1). Water soaking at the inoculation point was recorded but not considered as positive inoculation.

Some of the plant accessions did not develop citrus canker to any of the set of tested bacterial strains of *Xcc* and *Xca*. Leaves of the following species did not show any formation of hyperplastic tissue at the inoculation sites (Table 3): *Aegle marmelos*, *Calodendrum capense*, *Clausena excavata*, *Clausena lansium* (accessions both from CREA and CIRAD), *Fortunella margarita* (as described above), *Glycosmis pentaphylla*, and *Murraya paniculata*. Leaves of *A. marmelos* inoculated with *Xcc* C40 (A) and *Xca* JV596 (C) showed small water soaking areas around the inoculation sites (scored as ‘ws’).

All seven strains, regardless of the pathotype, induced the formation of hyperplastic tissues at the inoculation sites on *Eremocitrus glauca* (3 accessions), *Murraya ovatifoliolata*, and as described above, the reference *Citrus aurantiifolia*. Meanwhile, *Casimiroa edulis*, *Citrus limonia*, *C. microcarpa*, *C. paradisi*, *C. wintersii*, *F. margarita*, *F. japonica*, *M. koenigii*, Citrangequat, Faustrimedin, and Limequat Lakeland were resistant to the B and C *Xca* strains.

In addition, *C. edulis*, *C. limonia*, *C. microcarpa*, *M. koenigii*, *F. japonica*, Citrangequat, and Faustrimedin did not show any symptoms when inoculated with the *Xcc* A* and A^w^ strains. Mild reactions were observed on *Balsamocitrus dawei*, *C. myrtifolia*, *F*. *japonica* (amongst pathotype A strains only with C40), and *Atalantia disticha*. When inoculated with *Xcc* A or *Xca*, both or one of the two pathotypes, B and C, but not with A* and A^w^.

With regards to pathotype-based evaluation of pathotype A strains, 26 out of the 35 accessions tested were susceptible to at least one of them. Amongst pathotype A strains, *Xcc* C40 showed the widest panel of host reactions.

**Table 3:** Interaction phenotype between ornamental species of Rutaceae species and reference plants with bacterial strains *Xanthomonas citri* pv. *citri* and pv. *aurantifolii* ^1^.

Accessions ^2^	Bacterial Strain (Pathotype)						
	C40 (A)	LE116-1 (A)	LG97 (A)	LD71A (A*)	LG115 (A^W^)	JJ159 (B)	JV596 (C)
*Aegle marmelos*	ws	0	0	0	0	0	ws
*Atalantia buxifolia*	++	ws	0	ws	ws	ws	++
*Atalantia buxifolia (SRA746)*	++	+	+	+	+	+	+/++
*Atalantia ceylanica*	++	+	+	+	+	++	++
*Atalantia disticha*	+	0	0	ws	ws	+	++
*Balsamocitrus dawei*	+/++	+	+	0	0	+	+
*Calodendrum capense*	0	0	0	0	0	0	0
*Casimiroa edulis*	++	0	++	0	0	0	0
Citrangequat cv. Thomasville *	+++	+	+++	0	0	0	0
Citrus aurantiifolia **	+++	++++	++++	++++	+++	++	++++
*Citrus limonia* Osbeck var. *limonia*	+++	++	++++	0	0	0	0
*Citrus microcarpa*	++	+	++	0	0	0	0
*Citrus myrtifolia*	++++	+	++++	0	ws	++++	++++
Citrus paradisi **	++++	++	+++	++	0	0	0
*Citrus wintersii*	++++	++	0	++	0	0	0
*Clausena excavata*	0	0	0	0	0	0	0
*Clausena lansium* (CREASSGCF6P2)	0	0	0	0	0	0	0
*Clausena lansium* (SRA1080)	0	0	0	0	0	0	0
*Eremocitrus glauca* (CREASSGCF8P1)	++++	++	++++	++	++	++++	++
*Eremocitrus glauca* (SRA1001)	++++	++++	++++	+	+	+++	++/+++
*Eremocitrus glauca* (SRA871)	++++	++++	++++	+	+	++++	++/+++
Faustrimedin ^3^	++	++	++		0	0	0
*Fortunella hindsii*	+	++	++++	0	++	0	0
*Fortunella japonica*	+	0	0	0	0	0	+
*Fortunella margarita* (SRA490)	0	0	0	0	0	0	0
Fortunella margarita **	0	0	0	0	0	0	0
*Fortunella obovata*	+	0	0	0	0	0	+
*Glycosmis pentaphylla*	0	0	0	0	0	0	0
Limequat Lakeland *	++	0	0	+	0	0	0
*Microcitrus australasica* (SRA1002)	++	+	+	+	+	+	+
*Microcitrus australis*	++	ws	ws	0	+	+++	0
*Murraya koenigii*	++	0	0	0	0	0	0
*Murraya ovatifoliolata*	++++	+	+++	++	++++	++++	+
*Murraya paniculata*	0	0	0	0	0	0	0

^1^ Host–bacterial strain interaction was assessed visually, and scored using a scale as follows: 0, no symptoms as per water (negative control) inoculations, usually wound repair, and/or necrosis; ws, water soaking margin surrounding the wound sites; +, swelling of cell evident at site of inoculation (pustule or blister-like lesions); ++, beginning of callus formation or crystalline callus at inoculation site; +++, abundant crystalline callus (5 sites); ++++. confluent crystalline callus in a single hyperplasia. ^2^ Plants tested are in alphabetical order. Accessions without number are from CREA, Italy according to Table 2. The numbers are reported for accessions of plant species tested also at CIRAD, La Reunion, France that have the prefix SRA (specify). * hybrids; ** reference hosts.

### 3.3. Strain Virulence and Host Resistance Assessment

The results show that some species exhibited either no symptoms, or only weak reaction to inoculation with *Xcc* and *Xca*, ranging from water soaking to little pustule (blister-like) formation, or a little hyperplastic tissue, sometime crystalline at the inoculation sites (scored as ++). In this group of species, the formation of pustules in *F. japonica* and *F. obovata* was observed on the leaves inoculated with *Xcc* C40 (A lineage 1) and *Xca* JV596 (C) (Table 3); *M. koenigii* showed pustules and the beginning of callus formation when inoculated with the pathotype A strain *Xcc* C40; *A. buxifolia* CREASSVC and *A. disticha* showed callus formation only when inoculated with *Xcc* strain C40 (A) and *Xca* strain JV596 (C) and pustules or water soaking when inoculated with all the other strains except for *Xcc* LG97 for which no reaction was observed. Another accession of *A. buxifolia* (SRA746) showed pustules formation also with all other strains (Table 3). *B. dawei* showed a weak reaction following inoculation with all strains (pustules/blister like lesions) except for strains of A* and A^w^ pathotypes, which did not induce any symptoms. Additionally, in this group *C. edulis* showed the formation of crystalline callus at the sites inoculated with *Xcc* strain C40 (A lineage 1) and *Xcc* LG97 (A outlier), while Limequat Lakeland was positive to inoculation with *Xcc* C40 (A lineage 1) and *Xcc* LD71A (A*).

*E. glauca* and *M. ovatifoliolata* proved susceptible to all seven strains, and in some combinations formed a large crystalline callus in which the five inoculation sites coalesced (scored as ++++; Table 3). Minor symptoms were observed on *M. ovatifoliolata* leaves inoculated with *Xcc* LE116 (A lineage 2) and *Xca* strain JV596 (C).

All susceptible plants in the genus *Citrus* showed severe symptoms (mostly rated +++ or ++++) (Table 3). In particular, *C. limonia* and *C. microcarpa* and the trigeneric hybrid Citrangequat cv. Thomasville showed symptoms following inoculation only with pathotype A strains. Plants for which an interaction phenotype rated a +++ or ++++ included *F. hindsii* and *M. australis*.

*C. aurantiifolia* was highly susceptible to all *Xcc* and *Xca* strains, whereas *C. myrtifolia* did not show any symptom following inoculation with A* and A^w^ pathotype strains. The other *Citrus* species were susceptible to all A strains and *C. paradisi* and *C. wintersii* also to A* strain. Interestingly, the three *Murraya* species showed different interaction phenotypes with *M. ovatifoliolata* susceptible and *M. paniculata* resistant to *Xcc* and *Xca*, whereas *M. koenigii* was only susceptible to *Xcc* C40 (A).

Of the four accessions of the ornamental species belonging to the *Fortunella* genus, only *F. margarita* (two accessions) did not show any symptoms upon inoculation with all the strains, meanwhile, *F. hindsii*, *F. obovata*, and *F. japonica* showed variable reactions depending on the inoculated strain. A compatible reaction phenotype was observed in *F. hindsii* inoculated with strains assigned to A and A^W^ pathotypes; severe callus-like formation was observed with *Xcc* LG97 strain (A outlier). Leaves of the accession of *F. japonica* and *F. obovata* showed blister-like lesions when inoculated with *Xcc* C40 (A lineage 1) and *Xca* JV596 (C) strains. *Atalantia* spp. showed more severe symptoms when inoculated with *Xcc* C40 (A lineage 1) and *Xca* JV596 (C) pustule-like formation at the inoculation site, water soaking, or no symptoms were observed with all the other strains.

### 3.4. Bacterial Population Sizes

Bacterial population size was assessed on a semi-selective medium from leaf disks from 13 plant accessions sampled at the inoculation sites 7 dpi (Table 4). At the sampling date, most inoculation sites were still asymptomatic. Culturable *Xcc* or *Xca* populations were recovered on all assayed host species-strain combinations. While some values suggested that the inoculated strain(s) survived but they did not markedly multiply, some others clearly showed signs of bacterial multiplication. It is noteworthy that at this sampling time no evident correlation between disease development and bacterial population size was recorded. However, *E. glauca*, *F. hindsii*, *C. wintersii*, and *M. ovatifoliolata* later developed symptoms with most of the strains and also had the highest bacterial population sizes.

Likewise, *G. pentaphylla* and *C. lansium* supported the lowest mean bacterial population sizes and no symptoms developed with any of the strains. The same did not occur for *C. excavata* and *M. paniculata*.

ANOVA for each plant accession inoculated with the seven strains showed that, with few exceptions, the lowest bacterial population sizes were recorded for either or both strains *Xca* JJ159 (pathotype B) and *Xca* JV596 (pathotype C). No significant differences were found for the seven bacterial strains population sizes on *F. japonica*, *A. buxifolia*, *E. glauca*, and *C. wintersii*.

## 4. Discussion

Citrus bacterial canker affects most commercial varieties of citrus, limiting citrus production worldwide. *Xcc* enters host plant tissues through stomata or wounds and multiplies in the mesophyll. The main symptoms are formation of erumpent, and callus-like lesions with a water-soaked margin on leaves, fruits, and stem tissue. Severe attacks result in extensive defoliation, premature fruit dropping, and twig dieback [33]. Although the disease is circumscribed generally to *Citrus* spp., it may also occur in other species of the Rutaceae family.

Despite the importance of the disease, there is limited information available on comparative susceptibility among *Citrus* species and relatives to CBC. Furthermore, much of the available information was published prior to the identification of different strains of *Xcc* and *Xca*, produced using only a single strain of the bacteria, or evaluated under field conditions where one strain was assumed to be predominant. A further limiting factor is that rutaceous species are frequently used as ornamentals but have seldom been adequately screened to determine their host status. These rutaceous ornamentals are extensively grown in Mediterranean countries where CBC is not present, including in nurseries, orchards, private gardens, and public avenues and squares. Therefore, these plant species could increase the risk of citrus canker bacteria being transferred to CBC-free areas such as the EU and Mediterranean Basin. Importation of plants has caused CBC outbreaks in other parts of the world [34,35,36]. Therefore, deficiencies in phytosanitary controls, especially for non-regulated species such as non-citrus Rutaceae species, could allow citrus canker bacteria to be introduced into the EU and Mediterranean countries.

Accumulated evidence from natural and artificial infection with *X. citri* from different studies, including this one, clearly shows that bacterial canker is not strictly limited to the *Citrus* genus, but instead has a large number of hosts among the Rutaceae, certainly wider than the 23 species belonging to 20 Rutaceae genera reported in the past [9].

In terms of potentially immune species, Lee et al. [9] reported that no symptoms of bacterial canker had ever been observed on *A. buxifolia*, *A. marmelos*, *Balsamocitrus gabonensis*; *Xanthoxylum rhetsa*, or *Triphasia trifolia* [9]. In field observations, *M. paniculata*, *A. disticha*, and *F. japonica* were shown to be strongly resistant to CBC. Even on *G. pentaphylla* exposed in places where bacterial canker infection is easily possible, the disease was not observed [9]. *Swinglea glutinosa* showed naturally CBC infections with a lower susceptibility than that of sweet orange. Strong CBC infections were reported for *F. hindsii* [9]. The abundance of cankers found on *F. hindsii* trees supports the theory that this species may be a wild-born host from which CBC may spread into cultivated species [9].

In this study, for the first time a complete set of strains belonging to *Xcc* and *Xca* (representing the 5 describer pathotypes) were used to assess the interaction phenotype across a wide panel of ornamental species in the Rutaceae. In addition, three different strains (belonging to different lineages) of the most widespread pathotype A were fully characterized on 32 accessions of Rutaceae [15,22]. Our results with Rutaceae and pathotype A are mostly consistent with past findings, although some discrepancies were noted. For instance, in our experiments no symptoms were induced on *F. japonica* with most of the strains tested, as previously reported [9], but strains *Xcc* C40 (A) and *Xca* JV596 (C) produced slight symptoms. Moreover, Peltier and Frederich [37] stated that three species of *Microcitrus* and *Eremocitrus glauca* were susceptible to CBC, and in our study, this is fully confirmed. *Fortunella hindsii* was reported to be more vulnerable in greenhouses than three other Kumquat species, and for instance, symptoms in *F. japonica* and *F. margarita* plants only occurred in greenhouses. These two species were resistant in the field, with only mild reactions under optimum disease condition, with a high inoculum dose and physical wounding of the plant [25,38,39,40,41]. Our work with *Fortunella* spp. is in agreement with such results. Since *F. hindsii* was susceptible to most strains, *F. japonica* could be weakly infected with two of the strains, and no symptoms were induced in two accessions of *F. margarita*.

No symptoms were reported after inoculation of *G. pentaphylla* confirming previous observations by Peltier and Frederich [37]. Likewise, no symptoms were shown on two accessions of *C. lansium*, although small lesions have been described under greenhouse conditions by other authors [37]. *C. edulis* was previously reported as susceptible under greenhouse and field conditions, however, our results only partially confirm susceptibility because of the existence of crystalline callus at inoculation site with *Xcc* C40 (A) and *Xca* JJ598 (C) strains.

Hybrid species were also analyzed in our work and previously by Peltier and Frederich [37]. These authors observed that Faustrimedin and Faustrimon were more susceptible than the parent *Microcitrus australasica*. Faustrimedin was not so susceptible, with the infection restricted to small spots on the leaves and occasionally on twigs. All crossings of *Poncirus trifoliata*, including citranges, citrumelo, citradia, citrandarin, citrunshu, citrange, and citraldin produced susceptible offspring.

Our analysis was not restricted to symptomatology but also assessing the bacterial population in a range of hosts, to infer the bacterial interaction with the plant. In our conditions, all the strains could be detected in every plant species tested 7 dpi. However, bacterial populations were generally higher in those species found to be more susceptible to CBC according to symptomatology. For instance, *G. pentaphylla* and *C. lansium* did not show any symptoms and had the lowest bacterial population, whereas *M. ovatifoliolata*, *C. wintersii*, and *F. hindsii* had strong symptoms and high numbers of bacteria. The fact that bacteria could be isolated from plants where no symptoms ever developed can be explained by the short period of time (7 dpi) and the high bacterial concentration used.

Citrus canker bacteria survival in non-host plant has been described already on plant surfaces [40] and within the inoculated leaves [25,38,39]. In these, studies very high bacterial concentrations 7 dpi were observed. In particular, Chen et al. [38] observed that the numbers of *Xcc* recovered from non-necrotic infection areas among the 3 citrus plants with susceptible or resistant phenotypes were not significantly different when the leaves were inoculated with *Xcc* at 10^8^ cfu mL^−1^. Therefore, our study suggests caution when assessing host interactions based solely on bacterial population size. At lower bacterial titer, a reduction of the population size is, in fact, expected over time when an incompatible interaction occurs [25,38,39].

It is important to point out the high variability in symptoms among the different strains in the different hosts, which indicates a complex host–pathogen interaction for CBC. Our results confirm the importance of testing a large collection of strains in order to determine the susceptibility of a host, and the need to include in such studies representatives of all possible variants of the bacteria. Recently, a new method to rapid evaluate *X. citri* pv. *citri* titer has been developed. This method, based on an eYFP labeled *Xcc* strain, allowed the evaluation of citrus resistance to CBC and eliminated the necessity for plating on agar media [41]. This method, by avoiding the time-consuming plating approach, could allow a wider panel of germplasm to be screened using a set of representative strains, such as those used in our study, as well as test inoculation with different inoculum titer. Overall, the detached leaf assay has the advantage of miniaturization that makes possible the assessment of a large number of plant–strain combinations; it allows screening to occur in a confined environment with a quarantine pathogen. However, it should be kept in mind that environmental conditions prevailing in detached leaf assays are highly favorable to lesion production (presence of wounds, plant material at the most susceptible growth stage, optimal temperature, ~100% relative humidity) and thus represent an upper threshold for host status determination. For example, strain *Xcc* C40, a pathotype A (lineage 1) strain isolated from Réunion Island, produced callus-like reactions on *M. koenigii*, but this species has never been reported to host citrus canker lesions under field conditions in Réunion Island (O. Pruvost, unpublished data). These results are not strictly in contrast as for example the genetic background of the genotypes tested was unknown. Similar results were obtained in a resistance assessment when a pathotype A strain was inoculated at 10^8^ cfu mL**^−^**^1^ and 68% of the inoculated spots on kumquat leaves exhibited visible symptoms, although with smaller and flatter lesions than those that were observed on Mexican lime leaves [38]. Once more, this result highlights that further deep analysis needs to be performed to assess the full resistance of a species. The use of molecular markers based on pathogen-associated molecular pattern-triggered immunity responses, as those developed for *Citrus* species [42], which could be used in the future as a tool to help the process. Genome sequencing might also unravel the difference between accessions and resistance genetic determinants.

In Europe, ornamental citrus in pots is becoming increasingly popular and represents an activity of considerable interest. While until now commercial trade in ornamental potted citrus was mostly limited to lemon, calamondin, and kumquats, nowadays a considerable amount of ornamental breeding is taking place in many parts of the word. *Citrus* relatives are also being used in breeding programs to capture unique sources of resistance in conventional species of commercial interest. For example, *E. glauca* and *Microcitrus* spp. have recently been identified as potential sources of resistance to the devastating disease HLB, associated with the bacteria ‘*Candidatus* Liberibacter asiaticus’, including the possibility that *E. glauca* may offer full resistance [43]. Therefore, germplasm resistant to *X. citri* pv. *citri* is an important consideration in the breeding of both ornamental and conventional citrus types, whether it be via conventional approaches or biotechnological approaches, such as CRISPR mediated genome editing [44,45,46].

Therefore, it seems possible that trade will diversify into a wider range of species, including ornamental Rutaceae genera other than *Citrus*, with the risk of neglecting their potential as hosts of CBC. Failure to properly regulate the importation of Rutaceae species represents a possible pathway to unwittingly introduce and spread CBC to citrus-growing regions of Europe and the Mediterranean Basin. Our work supports the need to regulate the non-citrus Rutaceae not yet included in the Directive by the EU in order to keep the region free of this important pathogen for the citrus industry.

## Figures and Tables

**Table 1:** *Xanthomonas citri* pv*. citri* and pv. *aurantifolii* used in this study.

Strain ID	Pathovar	Pathotype	Host	Year of Isolation	Origin	Genetic Lineage ^a^	Strain Isolator
C40	*citri*	A	*C. sinensis*	1988	Réunion	1	CIRAD
LE116-1	*citri*	A	*C. aurantiifolia*	2008	Mali	2	CIRAD
LG97	*citri*	A	*C. limon*	2006	Bangladesh	outlier ^b^	FERA, UK
LG115	*citri*	Aw	*C. aurantiifolia*	2007	India	3	FERA, UK
LD71A	*citri*	A*	*Citrus* sp.	2007	Cambogia	4	CIRAD
JJ159	*aurantifolii*	B	*C. limon*	1988	Argentina	NA	USDA, USA
JV596	*aurantifolii*	C	*C. aurantiifolia*	1981	Brazil	NA	USDA, USA

NA: not available; ^a^ According to Pruvost et al. [22]. ^b^ According to Gordon et al. [15].

**Table 4:** *Xanthomonas citri* pv. *citri* and *X. citri* pv. *aurantifolii* bacterial population size (Log cfu mL*^−^*^1^) estimated at the inoculation point of detached leaves of representative ornamental rutaceous plants ^1^.

Plant Species	C40 (A)	LE116 (A)	LG97 (A)	LD71A (A*)	LG115 (A^W^)	JJ159 (B)	JV596 (C)
*Glycosmis pentaphylla*	4.25 b	4.62 b	4.02 b	4.08 b	4.11 b	2.52 a	3.82 b
*Clausena lansium*	4.17 b	6.28 d	5.37 c	4.89 c	3.52 b	1.67 a	1.67 a
*Murraya koenigii*	4.58 ab ^2^	5.55 b	5.01 b	5.44 b	3.76 a	4.00 a	4.06 a
*Fortunella japonica*	3.52 a	4.67 a	5.26 a	5.46 aba	4.30 a	5.04 a	4.42 a
*Clausena exavata*	5.36 b	6.06 b	5.32 b	5.49 abc	6.08 b	2.87 a	5.36 b
*Fortunella obovata*	5.79 d	6.39 e	5.34 c	5.19 c	6.23 de	4.52 b	3.82a
*Atalantia buxifolia*	5.21 a	5.89 a	4.67 a	6.44 a	6.08 a	4.40 a	5.15 a
*Murraya paniculata*	6.16 b	6.05 b	5.95 b	5.79 b	5.55 b	4.43 a	5.63 b
*Atalantia disticha*	5.37 a	6.23 b	6.35 b	5.00 a	6.13 b	6.23 b	7.36 c
*Eremocitrus glauca*	5.32 a	7.30 a	7.39 a	7.08 a	5.54 a	4.56 a	6.15 a
*Fortunella hindsii*	9.22 c	9.65 c	6.32 b	9.73 c	5.40 ab	6.24 b	5.00 a
*Citrus wintersii*	7.06 a	8.38 a	7.53 a	9.52 a	8.27 d	5.91 a	6.91 a
*Murraya ovatifoliolata*	7.29 b	7.31 b	7.49 b	8.45 b	9.35 c	6.14 a	8.27 b

^1^ Means followed by the same letter in the rows are not significantly different at *p* ≤ 0.01 according to the Student–Newman–Keuls test. ^2^ The presence of pustules (+) or water soaking as results of the inoculation was highlighted by light gray boxes; phenotype interactions which resulted in symptoms recorded as ++, +++, and ++++ in dark grey; all the other inoculations did not show any symptoms according to Table 3.

## Data Availability

Not applicable.
